# Bioactivity-Guided Fractionation of the Bidah Pomegranate Landrace Identifies a Bioactive Fraction Inducing Mitochondria-Associated Apoptotic Responses in Colorectal Cancer Cells

**DOI:** 10.3390/ijms27062808

**Published:** 2026-03-20

**Authors:** Saheed O. Anifowose, Nada M. Alattas, Khalid M. AL-Rohily, Abdalrhaman M. Salih

**Affiliations:** 1 National Research and Development Center for Sustainable Agriculture (Estidamah), Riyadh Techno Valley, Riyadh 12373, Saudi Arabia or 444105896@student.ksu.edu.sa (S.O.A.); alattas@estidamah.gov.sa (N.M.A.); khalb6@gmail.com (K.M.A.-R.); 2 Zoology Department, College of Science, King Saud University, Riyadh 11451, Saudi Arabia; 3 Botany and Microbiology Department, College of Science, King Saud University, Riyadh 11451, Saudi Arabia

**Keywords:** Bidah pomegranate, sustainable medicinal plants, intrinsic apoptosis, antioxidant phytochemicals, chromatographical analysis, Saudi Arabia cultivar

## Abstract

Pomegranate (*Punica granatum* L.) has attracted considerable attention for its anticancer potential; however, mechanistic studies employing bioactivity-guided fractions from geographically distinct landraces remain limited. Building on our previous report on the bioactivity and phytochemical profile of the Bidah pomegranate landrace, the present study applied bioactivity-guided fractionation to enrich biologically active constituents and investigate mitochondria-associated cellular responses in colorectal cancer cells (Caco-2 cells). A semi-polar fraction from Bidah pomegranate crude extract (B6) was evaluated for its antioxidant activity, cell viability, cell death morphology, mitochondrial membrane potential, transcriptional modulation of key regulatory genes, and phytochemical composition. High-performance liquid chromatography (HPLC) profiling of B6 revealed a chromatographic fingerprint with seven detectable peaks, including two major peaks at retention times of 7.577 and 8.602 min, together accounting for approximately 66% of the total chromatographic area, indicating the enrichment of major constituents. Consistent with this enrichment, the fraction exhibited potent DPPH radical scavenging activity at a microgram-range IC_50_, suggesting the presence of redox-active phytochemicals. In cell-based assays, the fraction induced a dose-dependent reduction in metabolic viability, while acridine orange/propidium iodide (AO/PI) staining of Caco-2 cells revealed delayed, regulated cell death. JC-1 staining demonstrated a pronounced loss of mitochondrial membrane potential, consistent with early mitochondrial dysfunction. Gene expression analysis further revealed modulation of pro- and anti-apoptotic genes, alongside cell-cycle-associated and oxidative stress/inflammatory markers. Gas chromatography–mass spectrometry (GC–MS) profiling identified polyacetylenes, sterol derivatives, fatty acid esters, and terpenoids, providing chemical context for the observed mitochondrial perturbation. Collectively, the findings support a mitochondria-centered, regulated cell death response driven by a multi-component phytochemical matrix. This study advances mechanistic insight beyond crude extract analysis and highlights the sustainable biomedical value of the Bidah pomegranate landrace as an underutilized regional resource.

## 1. Introduction

The global incidence of cancer-related morbidity and mortality remains alarmingly high. According to the GLOBOCAN 2022 report [1], colorectal cancer ranks as the third most diagnosed malignancy worldwide, accounting for approximately 10% of all new cancer cases [2]. Cancer is a multifactorial disease arising from genetic instability, which leads to the accumulation of loss- and gain-of-function mutations that disrupt critical cellular regulatory processes and compromise cellular homeostasis [3]. Among these alterations, dysregulation of the apoptotic machinery represents a fundamental driver of colorectal cancer progression and therapeutic resistance [4]. Aberrant balance between pro- and anti-apoptotic signaling promotes malignant cell survival under stress conditions [5], while oxidative stress imbalance and defective cell death signaling further contribute to tumor persistence and disease progression [6]. Recent and sustainable advances in drug discovery have led to the development of contemporary chemotherapeutic strategies, resulting in measurable improvements in patient outcomes [7,8]. However, the clinical efficacy of these therapies is frequently constrained by off-target toxicity and the emergence of drug resistance, thereby leading to the urgent need for pleiotropic agents capable of modulating multiple intracellular signaling pathways with improved therapeutic selectivity [8].

Bioprospecting medicinal plants for bioactive secondary metabolites has long been a cornerstone of anticancer drug discovery, owing to their structural diversity and favorable biological activity [3,9]. Numerous phytochemicals have been shown to modulate redox homeostasis, cell cycle progression, and programmed cell death pathways in cancer cells [10]. *Punica granatum* L. exhibits a well-documented bioactivity profile attributed to its richness in polyphenols, ellagitannins, flavonoids, and fatty acid derivatives [11]. In our recent study of the Bidah pomegranate landrace, the crude extract demonstrated notable antioxidant capacity, alongside antiproliferative activity against colorectal cancer cells [12]. However, the chemical complexity of crude extracts complicates their mechanistic interpretation due to overlapping and potentially antagonistic interactions among constituents [13]. To address this limitation, bioactivity-guided fractionation was employed to enrich biologically relevant components and improve mechanistic clarity. The present study therefore investigates the mechanistic effects of a bioactivity-guided semi-polar fraction from the Bidah pomegranate landrace in colorectal cancer cells, integrating cytotoxic profiling with cellular morphology assessment and early markers of cell death to establish a mechanistically informed foundation for subsequent compound isolation and molecular pathway elucidation.

## 2. Results

### 2.1. Bioactivity-Guided Fractionation of Bidah Pomegranate Extract

**Figure 1:**
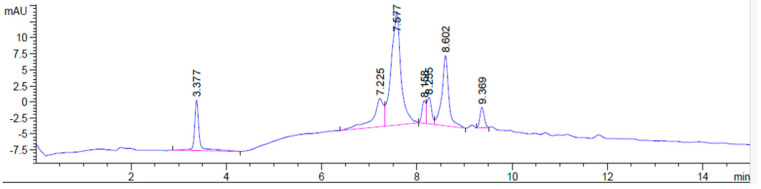
HPLC chromatogram of the B6 bioactive fraction indicating enrichment of secondary metabolites.

The silica gel column chromatography of the crude extract of the Bidah pomegranate landrace yielded seven fractions. The fraction obtained in an ethyl acetate/methanol 1:1 gradient solvent showed the enrichment of secondary metabolites, as it exhibited distinct TLC bands when stained with the general stain, p-anisaldehyde. HPLC profiling of this fraction revealed a simplified chromatographic profile with seven detectable peaks, indicating the enrichment of secondary metabolites following bioactivity-guided fractionation (Figure 1).

Table 1 shows that two major peaks were observed at retention times of 7.577 min (45.66% of total peak area) and 8.602 min (20.00% of total peak area), together accounting for approximately two-thirds of the total chromatographic area of the B6 fraction. Additional minor peaks were detected at retention times of 3.377, 7.225, 8.158, 8.255, and 9.369 min. These findings confirm that the B6 fraction contains a limited number of enriched constituents that may collectively contribute to its anticancer activity. This fraction was therefore selected for subsequent bioactivity validation and mechanistic investigations, and is hereafter referred to as B6.

### 2.2. Effects of B6 Fraction on Antioxidant Activity and Caco-2 Cell Viability

**Figure 2:**
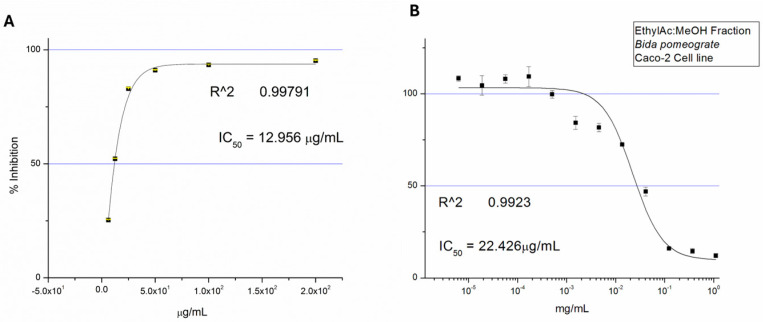
(**A**) DPPH scavenging activity (%) of DPPH radical scavenging activity and (**B**) MTT-based cell viability of B6 fraction against Caco-2 cells. The cell viability was assessed in a concentration-dependent manner. Data are expressed as mean ± SD (*n* = 3).

The biological activity of the B6 fraction was evaluated through assessment of both antioxidant capacity and cell viability assay against Caco-2 cells. Figure 2A shows that the fraction exhibited potent antioxidant activity in the DPPH radical scavenging assay, yielding an IC_50_ value of 12.956 µg/mL. Furthermore, the MTT assay (Figure 2B) revealed a dose-dependent reduction in Caco-2 cell viability with an IC_50_ of 22.426 µg/mL. These results indicate that the bioactivity-guided fractionation enriched the B6 fraction with bioactive constituents, while other fractions possessed low or no bioactivity in Caco-2 cells, with IC_50_ values above 100 µg/mL.

### 2.3. B6 Fraction Reveal Morphologically Delayed Apoptotic Cell Death

**Figure 3:**
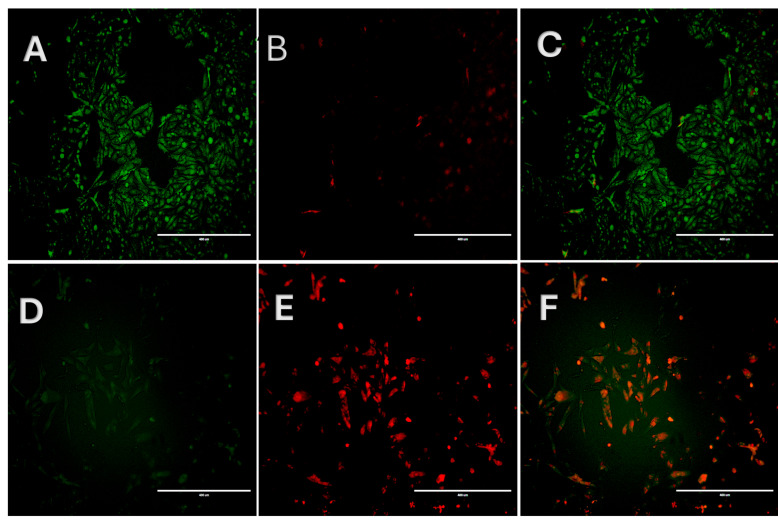
Acridine orange/propidium iodide (AO/PI) staining of Caco-2 cells treated with the B6 fraction from the Bidah pomegranate landrace. Scale bar: 400 µm. Photomicrographs (**A**–**C**) represent vehicle-treated cells displaying predominantly AO-positive (green fluorescence) and PI-negative staining; this indicates viable cells with intact membranes. Photomicrographs (**D**–**F**) represent B6-treated cells displaying increased PI uptake (red flourescence) and reduced AO staining; this is consistent with loss of membrane integrity and apoptotic-like cell death.

Acridine orange/propidium iodide (AO/PI) staining was performed to examine morphological changes and membrane integrity in Caco-2 cells following treatment with the B6 fraction (Figure 3). Photomicrographs A–C show methanol-treated cells. Photomicrograph A (green signal) shows uniformly stained nuclei after staining with AO, reflecting the intact nuclear morphology; in addition, the PI-stained cells show negligible cellular uptake, indicating preserved membrane integrity and cellular viability. In contrast, in B6-treated cells at 50 µg/mL (photomicrographs D–F), AO/PI staining suggests apoptotic commitment. This is evidenced by the loss of AO signal (AO intercalate double-strand DNA), nuclear condensation, and consequent cytoplasmic PI uptake. The presence of PI after nuclear morphological changes indicates the loss of membrane integrity subsequent to apoptotic commitment. Merged AO/PI images (Panel F) revealed a heterogeneous population of AO- and PI-positive cells, consistent with a progressive transition from early cellular stress to late cell death. The absence of widespread uniformly PI-positive rounded cells suggests that cell death occurred through a regulated apoptotic process rather than acute necrosis.

### 2.4. JC-1 Staining Reveals Mitochondrial Membrane Depolarization in Treated Caco-2 Cells

**Figure 4:**
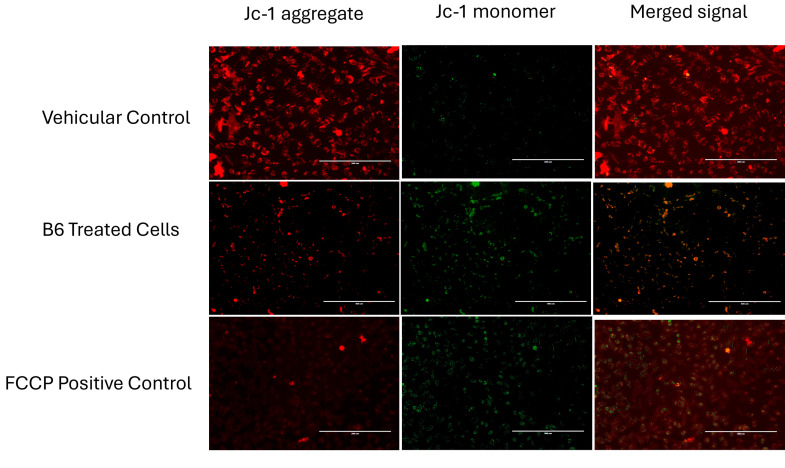
JC-1 staining of Caco-2 cells following treatment with B6 fraction from the Bidah pomegranate landrace. Red fluorescence indicates polarized (healthy) mitochondria, whereas green fluorescence represents depolarized mitochondria. Scale bars: 200 µm and 400 µm.

Staining of Caco-2 cells with JC-1 dye for determining the mitochondrial membrane potential (ΔΨm) was used to confirm the suggested phenotypic loss of membrane integrity in B6-treated cells. Figure 4 shows vehicular control cells with predominant red, fluorescent signal, indicating intact mitochondrial membrane potential. The FCCP-treated positive control shows a marked loss of red fluorescence signal and a corresponding increase in green fluorescence, indicating mitochondrial depolarization (Figure 4, FCCP panels). Similar to the positive control, B6-treated cells also demonstrated a pronounced red-to-green fluorescence shift (Figure 4, B6-treated panels), indicating disruption of ΔΨm. Meanwhile, the extent of depolarization was less pronounced than FCCP, indicating the partial loss of ΔΨm and mitochondrial dysfunction, consistent with early involvement of the regulated cell death pathway.

### 2.5. Modulation of Apoptosis-Related Gene Expression in Caco-2 Cells

**Figure 5:**
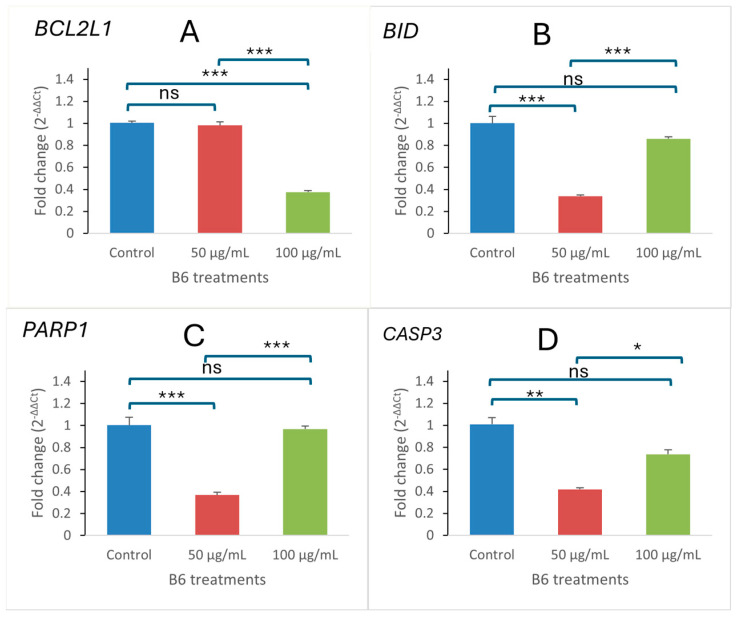
Quantification of relative mRNA expression levels of (**A**) *BCL2L1*, (**B**) *BID*, (**C**) *PARP1* and (**D**) *CASP3* after treatment at 50 and 100 µg/mL and normalized to the control group. Data are presented as mean fold change ± SD (*n* = 3). Statistical analysis was performed using one-way ANOVA followed by Tukey’s post hoc test. * *p* < 0.05, ** *p* < 0.01, *** *p* < 0.001; ns, not significant.

The gene expression profile of some pro- and anti-apoptotic genes was carried out using RT-qPCR analysis. Figure 5 shows a significant modulation of some apoptosis-related genes in B6-treated Caco-2 cells. Anti-apoptotic gene *BCL2L1* (Figure 5A) was found downregulated in a dose-dependent manner relative to the normalized control. In contrast, the intrinsic apoptosis regulator *BID* (Figure 5B) exhibited reduced expression relative to the untreated control following B6 treatment, although a concentration-dependent increase was observed between treatment doses. Similarly, *PARP1* and *CASP3* (Figure 5C,D), which are execution-phase apoptosis markers, remained below control levels but showed a significant dose-dependent increase in expression in treated cells. Collectively, the gene expression profile observed supports activation of the intrinsic apoptotic pathway and corroborates the morphological and mitochondrial dysfunction observed in complementary assays. These transcriptional changes are consistent with the mitochondrial membrane depolarization observed by JC-1 staining and the delayed apoptotic features detected by AO/PI analysis, supporting the involvement of mitochondria-associated apoptotic pathways.

### 2.6. Modulation of Cell-Cycle-Related Gene Expression in Caco-2 Cells

**Figure 6:**
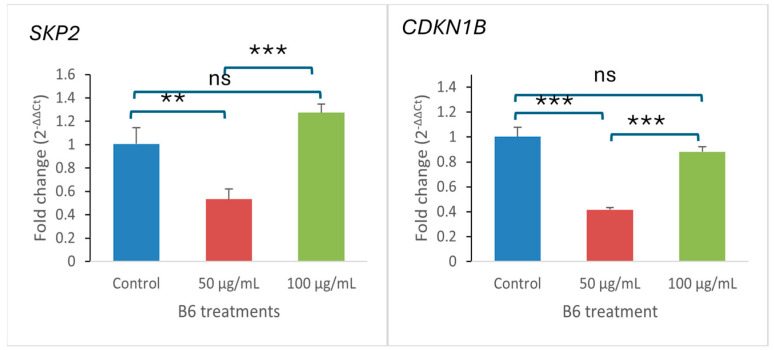
Relative mRNA expression levels of *SKP2* and *CDKN1B* were quantified after treatment at 50 and 100 µg/mL and normalized to the control group. Data are presented as mean ± SD (*n* = 3). Statistical analysis was performed using one-way ANOVA followed by Tukey’s post hoc test. ** *p* < 0.01, *** *p* < 0.001; ns, not significant.

After the gene expression profile of apoptosis-related genes suggesting the induction of intrinsic apoptosis, further gene expression analysis showed that the treatment of Caco-2 cells with the B6 fraction significantly altered the expression of key cell-cycle regulatory genes (Figure 6). *SKP2*, an oncogenic cell-cycle regulator was found to be regulated in a dose-dependent manner, with a marked reduction at 50 µg/mL followed by a pronounced upregulation at 100 µg/mL relative to the control sample. Meanwhile, the *CDKN1B* gene (cyclin-dependent kinase inhibitor) was found to be downregulated at 50 µg/mL, while it was slightly upregulated at 100 µg/mL. These findings indicate that B6 treatment disrupts cell-cycle regulatory balance, suggesting dose-dependent effects on proliferative control in colorectal carcinoma.

### 2.7. Modulation of Oxidative Stress and Inflammation-Related Gene Expression

**Figure 7:**
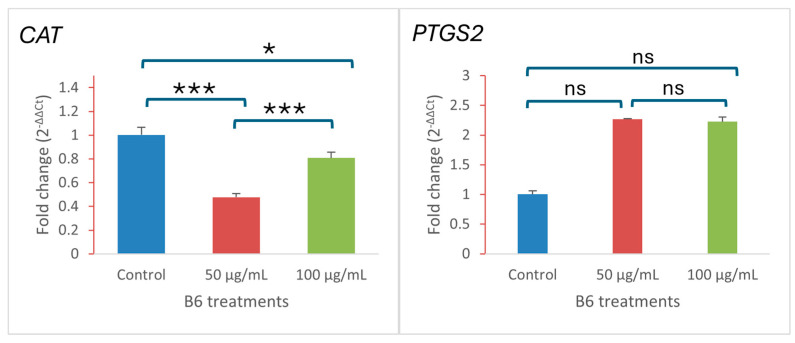
Quantification of relative mRNA expression levels of *CAT* and *PTGS2* after treatment at 50 and 100 µg/mL and normalized to the control group. Data are presented as mean ± SD (*n* = 3). Statistical analysis was performed using one-way ANOVA followed by Tukey’s post hoc test. * *p* < 0.05, *** *p* < 0.001; ns, not significant.

The combined morphological and molecular evidence supports a regulated mode of cell death involving stress adaptation and apoptotic signaling. Further supporting evidence from the RT-qPCR analysis of oxidative stress-related genes shows that B6-treated colon carcinoma cells altered the expression of these genes. Figure 7 shows that *CAT* (catalase), a key antioxidant enzyme, was markedly downregulated at both 50 and 100 µg/mL compared with control cells, indicating suppression of cellular antioxidant capacity. In contrast, the expression of cyclooxygenase (*PTGS2*) was strongly upregulated in treated cells, with a pronounced increase observed at both concentrations. This divergent expression pattern suggests the induction of oxidative and inflammatory stress signaling in response to B6 treatment.

### 2.8. GC–MS Identification of Bioactive Phytochemicals in B6 Fraction

**Figure 8:**
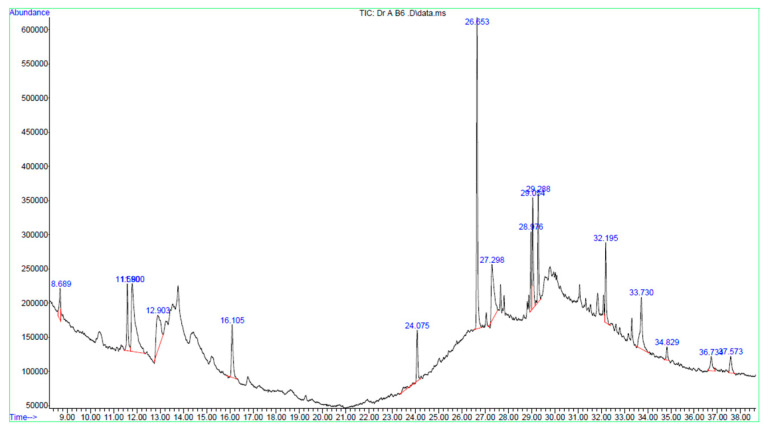
GC-MS chromatogram of B6 fraction obtained from the leaf extract of Bidah pomegranate landrace.

The GC–MS secondary metabolite profiling of B6 showed a complex phytochemical profile dominated by lipophilic compounds, including fatty acid derivatives, sterol-like compounds, terpenoids, and polyacetylenes (Table 2 and Figure 8). Among the identified constituents, several compounds with reported relevance to cancer biology and mitochondrial function were detected. Interestingly, falcarinol, a polyacetylene previously associated with anticancer activity, and estera-1,3,5(10)-trien-17β-ol, a steroid-like compound, were present at appreciable levels. In addition, the fraction contained abundant fatty acid methyl esters and unsaturated fatty acids, including methyl palmitate and oleic acid, which are known to influence cellular lipid homeostasis and mitochondrial membrane properties. The presence of terpenoid derivatives, such as cedran-diol, further contributes to the bioactive chemical landscape of the fraction.

Collectively, the GC–MS profile indicates enrichment of phytochemicals capable of interacting with cellular membranes and mitochondrial function, providing a chemical context for the observed mitochondrial membrane depolarization, reduced cell viability, and progression toward apoptotic cell death observed in cellular assays.

## 3. Discussion

Our previous investigation on the Bidah pomegranate landrace, a cultivar native to the Al Baha region of Saudi Arabia, demonstrated the broad antioxidant and anticancer potential, but the limited mechanistic resolution, of the crude extract [12]. This present study advances our understanding by applying bioactivity-guided fractionation to enrich both chemical and biological interpretation. The bioactivity-guided fractionation approach yielded a bioactive fraction, B6, that demonstrates a clearer linkage between phytochemical enrichment, mitochondrial dysfunction, and regulated cell death in colorectal cancer cells, while reducing the confounding effects inherent to complex crude extracts [20]. As reflected by a lower DPPH IC_50_, the B6 fraction demonstrated higher antioxidant efficiency relative to the crude extract. This is consistent with prior reports that fractionation of pomegranate matrices enriches bioactive lipophilic constituents with higher redox reactivity [21]. Notably, this antioxidant capacity coincided with reduced cellular metabolic activity and mitochondrial membrane depolarization, suggesting that redox-active compounds in the fraction may impose oxidative and metabolic stress in cancer cells [22]. This redox behavior has been reported for polyphenol-associated phytochemicals and several pomegranate-derived compounds, particularly under cancer-specific metabolic conditions [23]. Mitochondrial dysfunction emerged as a central convergence point across multiple assays in this study. JC-1 staining demonstrated mitochondrial membrane depolarization following B6 exposure, while AO/PI staining revealed delayed membrane permeability changes consistent with regulated cell death rather than acute necrosis. At the transcriptional level, several apoptosis-related genes showed modulation following treatment. The relative expression of *BID*, *PARP1*, and *CASP3* remained below untreated control levels but exhibited a concentration-dependent increase across treatment conditions. This pattern suggests a partial restoration toward apoptotic competence rather than direct transcriptional upregulation above baseline levels. In the context of mitochondrial apoptosis signaling, *BID* plays a key role in linking upstream death signals to mitochondrial outer-membrane permeabilization through cleavage into truncated BID (tBID), which promotes mitochondrial destabilization and cytochrome c release [24]. The observed reduction in *BID* transcription at a lower concentration may therefore reflect early mitochondrial engagement, mediated primarily through post-translational activation rather than de novo transcription. Interestingly, *BID* expression showed partial recovery at higher concentrations, potentially indicating compensatory transcriptional responses under intensified cellular stress [25]. This pattern coincided with modulation of the anti-apoptotic gene *BCL2L1* (encoding BCL-xL), supporting the notion that B6 treatment perturbs mitochondrial survival signaling and promotes mitochondrial outer-membrane permeabilization [26]. Additional modulation of *CASP3* and *PARP1*, key execution-phase apoptosis components, further suggests the engagement of regulated cell death signaling [27], although definitive pathway confirmation will require protein-level validation. Together, these findings indicate that B6 treatment disrupts mitochondrial stability and apoptotic regulatory balance rather than activating a single linear death pathway. The inclusion of additional markers such as *SKP2*, *CDKN1B* (p27), *CAT*, and *PTGS2* further suggests perturbation of proliferative control, oxidative stress regulation, and inflammatory signaling pathways, indicating broader disruption of cancer cell homeostasis.

GC–MS profiling provides chemical context for these biological effects, revealing the enrichment of fatty acid derivatives, sterol-like compounds, terpenoids, and polyacetylenes such as falcarinol, several of which have been independently linked to mitochondrial stress and apoptosis in cancer models [15,17]. It should be noted, however, that these constituents are tentatively assigned based on NIST library matching and require confirmation using authentic standards and orthogonal analytical approaches such as LC–MS/MS or NMR. Rather than attributing activity to a single compound, the present findings support a multi-component, membrane-centered mode of action consistent with phytochemical synergy, while definitive structure–activity relationships will require further purification and structural validation.

This study has delineated the potential of the Bidah pomegranate landrace to induce regulated cell death; however, the study is limited by the absence of protein-level validation of apoptotic execution and direct intracellular ROS measurements. Collectively, protein-level validation, including the assessment of cleaved CASP3, *PARP1* cleavage, and cytochrome c release, will be undertaken following further purification and structural elucidation of the active metabolite(s), as mechanistic profiling at the semi-purified fraction stage is intended to guide focused compound isolation and downstream target-validation studies. Subsequently, cross-validation in additional colorectal cancer cell lines and non-tumorigenic colon epithelial models will be performed to assess reproducibility and tumor selectivity of the observed mitochondria-associated apoptotic phenotype. Importantly, the Bidah pomegranate landrace represents a locally adapted, underutilized genetic resource. Demonstrating value-added biomedical potential through fractionation-based research supports its sustainable utilization, conservation, and potential integration into regional nutraceutical or pharmaceutical development pipelines.

## 4. Materials and Methods

### 4.1. Plant Material and Preparation of B6 Fraction

The dried leaves of the Bidah pomegranate landrace (*Punica granatum* L.) were originally collected from the Bidah region of Saudi Arabia. The plant was maintained in the micropropagation facility of National Research and Development Centre for Sustainable Agriculture (Estidamah), Riyadh, Saudi Arabia. The leaves were processed as previously described in our earlier study on the crude extract [12]. Dried leaves of Bidah pomegranate were ground and macerated in methanol (Sigma, St. Louis, MO, USA) to obtain the crude extract. Then, bioactivity-guided fractionation was subsequently performed on the crude extract using silica gel column chromatography (SGCC) with a gradient solvent system comprising hexane (100%), hexane/dichloromethane (50:50), dichloromethane (100%), dichloromethane/ethyl acetate (50:50), ethyl acetate (100%), ethyl acetate/methanol (50:50) and methanol (100%). Based on preliminary antioxidant and cytotoxic screening, the ethyl acetate/methanol fraction (B6) was selected for further biological and chemical evaluation.

### 4.2. HPLC Separation of the B6 Fraction

The bioactive fraction (B6) was subjected to high-performance liquid chromatography (HPLC) analysis using an Agilent Technologies 1290 Infinity system (Agilent Inc., Palo Alto, CA, USA) for chromatographic profiling and assessment of chemical enrichment. Separation was achieved on a ZORBAX RX-C18 analytical column (1.8 μm, 4.6 × 150 mm) using a HPLC-grade gradient elution system consisting of methanol (solvent A) and acetonitrile (solvent B). The gradient program was optimized to achieve an adequate resolution of major constituents. Detection was performed using a diode array detector (DAD) set at 280 nm. An injection volume of 5 µL of B6 solution (10 mg/mL) was used for each run. Chromatographic analyses were performed at ambient column temperature and repeated to confirm reproducibility of the chromatographic profile.

### 4.3. DPPH Radical Scavenging Assay

The antioxidant activity of B6 fraction was assessed using the DPPH free radical scavenging assay [28]. Briefly, a solution of 0.1 mmol L^−1^ DPPH in methanol was prepared. Thereafter, equal volumes of 750 μL of the B6 fraction and DPPH solution were added together. The mixture was incubated for 20 min at 25 °C, and the absorbance was measured at 517 nm.

Percentage radical scavenging activity was calculated relative to a control, and concentration–response curves were generated. IC_50_ values were determined by nonlinear regression analysis using Origin software (Version 6, OriginLab Corporation, Northampton, MA, USA).

### 4.4. Cell Culture and Cell Viability Assay

Caco-2 human colorectal adenocarcinoma cells (Caco-2 ATCC collection: Caco-2: HTB-37) were cultured in Dulbecco’s Modified Eagle Medium (DMEM) supplemented with 10% fetal bovine serum (FBS), 1% antibiotic–antimycotic (Gibco™, Grand Island, NY, USA) and maintained at 37 °C in a humidified incubator with constant 5% CO_2_ supply. Cells were sub-cultured at 70–80% confluence and used for experiments. Cell viability was evaluated using the MTT assay [29]. Caco-2 cells were seeded into 96-well plates. Cells were then treated with increasing concentrations of the B6 fraction for the indicated incubation period. Following treatment, the MTT solution was added and incubated to allow for the formation of formazan crystals, which were subsequently dissolved in isopropanol. Absorbance was measured at 570 nm, and cell viability was expressed as a percentage relative to untreated control cells. IC_50_ values were calculated using nonlinear regression analysis with Origin software (OriginLab Corporation, Northampton, MA, USA).

### 4.5. Acridine Orange/Propidium Iodide (AO/PI) Staining

To assess cellular morphology and membrane integrity, AO/PI dual staining was performed [30]. After 24 h of B6 treatment at 50 µg/mL, Caco-2 cells were co-stained with acridine orange and propidium iodide at 10 µg/mL, washed 2× with PBS and left in HEPES buffer for immediate observation under a fluorescence microscope. Green fluorescence indicated AO uptake in viable cells, while red fluorescence indicated PI uptake in cells with compromised membrane integrity. Images were captured using EVOS FL fluorescence microscope (Thermo Fisher Scientific, Waltham, MA, USA).

### 4.6. JC-1 Mitochondrial Membrane Potential Assay

Mitochondrial membrane potential (ΔΨm) was evaluated using the Abcam JC-1 fluorescent probe (ab113850, Abcam plc, Cambridge, UK). Caco-2 cells were incubated with the B6 fraction at 50 µg/mL for 24 h. FCCP was added as a positive control 2 h before the completion of B6 incubation period, while methanol served as the vehicular control. Post B6 treatment, the culture media were removed and washed twice with a supplemented buffer before being added to a pre-warmed JC-1 working solution (2 µM in assay buffer).

Stained cells were incubated at 5% CO_2_, 37 °C in the dark for 25 min. Thereafter, the cells were washed twice with the JC-1 assay buffer supplemented with 25 mM HEPES to stabilize pH during imaging. Fluorescent images were acquired immediately using an EVOS FL fluorescence microscope (Thermo Fisher Scientific, Waltham, MA, USA). JC-1 monomers were visualized using the green (FITC) channel (excitation/emission ~488/530 nm), while JC-1 aggregates were visualized using the red (TRITC) channel (excitation/emission ~540/590 nm). Exposure settings were kept constant across samples. The images were acquired promptly to minimize photobleaching and dye leakage.

Qualitative assessment of mitochondrial polarization was based on the red-to-green fluorescence pattern. Quantitative analysis was performed using ImageJ (1.8.0-431) software to measure the mean fluorescence intensity of red and green signals and to calculate the red/green fluorescence ratio as a proxy for ΔΨm status.

### 4.7. RNA Extraction and RT-qPCR Analysis

Total RNA was extracted from treated and control Caco-2 cells using a RNeasy extraction kit from QIAGEN GmbH, Hilden, Germany. Following the kit’s protocol, Caco-2 cells were cultured in 6-well plates and allowed to reach around 75% confluence before B6 treatment. Cells were treated at 50 µg/mL and 100 µg/mL for 24 h before the total RNA extraction was performed. RNA concentration and purity were checked via Nanodrop. Complementary DNA (cDNA) was synthesized using a High-Capacity cDNA Reverse Transcription Kit (Applied Biosystems, Thermo Fisher Scientific, Waltham, MA, USA) following the kit’s protocol.

Quantitative real-time PCR (RT-qPCR) was performed using SYBR Green master mix (Applied Biosystems, Thermo Fisher Scientific, Waltham, MA, USA) to evaluate the expression of apoptosis-related genes (*BCL2L1*, *BID*, *CASP3*, *PARP1*), cell-cycle-related genes (*SKP2*, *CDKN1B*), and oxidative stress/inflammation-related genes (*CAT*, *PTGS2*). Amplification was conducted on a QuantStudio™ real-time PCR system (Applied Biosystems, Thermo Fisher Scientific, Waltham, MA, USA) under standard cycling conditions. Melt-curve analysis was performed to confirm primer specificity. Gene-specific primers were synthesized by Macrogen Inc., Seoul, Republic of Korea. The primer information is provided in Table 3. Relative gene expression was calculated using the 2^−ΔΔCt^ method, with normalization to GAPDH.

### 4.8. GC–MS Analysis

Gas chromatography–mass spectrometry (GC–MS) was employed to profile the constituents present in the B6 bioactive fraction. An aliquot (2 µL) of the prepared B6 sample was injected into an Agilent 5977B gas chromatograph coupled with a mass selective detector (Agilent Technologies, Santa Clara, CA, USA). Chromatographic separation was performed using an HP-5MS Ultra Inert (UI) capillary column (30 m × 0.25 mm i.d. × 0.25 µm film thickness), with helium as the carrier gas, at a constant flow rate of approximately 1.0 mL/min. The oven temperature was initially held at 80 °C for 2 min, followed by a programmed increase to 300 °C to enable the elution of higher-boiling constituents. The total run time was 38 min. Mass spectral data were acquired in electron ionization mode at 70 eV, with the ion source and quadrupole temperatures maintained at 230 °C and 150 °C, respectively. A solvent delay of 7 min was applied, and mass spectra were recorded over an *m*/*z* range of 40–550. Data acquisition and processing were performed using Agilent MassHunter software (version 10.0). Compound identification was carried out by comparing the obtained mass spectra with those in the NIST 14.L mass spectral library.

### 4.9. Statistical Analysis

All experiments were performed in triplicate, and data are presented as mean ± standard deviation (SD). Statistical analyses were conducted using SPSS (version 20) software, and concentration–response relationships were evaluated by nonlinear regression using Origin software. One-way analysis of variance (ANOVA) was performed to compare the means, and the significance level was determined at (*p* < 0.05).

## 5. Conclusions

In conclusion, bioactivity-guided fractionation of the leaves of Bidah pomegranate landrace enhanced both chemical clarity and mechanistic insight beyond that achieved with the crude extract. The B6 fraction exhibited potent antioxidant activity, induced mitochondrial membrane depolarization, and promoted a regulated, non-necrotic mode of cell death in colorectal cancer cells. Transcriptional modulation of survival-, cell-cycle-, and stress-related genes, together with an enrichment of lipophilic phytochemicals such as polyacetylenes, sterol derivatives, and fatty acid esters, supports a mitochondria-centered biological response driven by multi-component phytochemical interactions. While further studies are required to validate apoptotic execution at the protein level and to isolate individual bioactive constituents, these findings demonstrate the therapeutic potential of the Bidah pomegranate landrace and highlight its sustainable value as an underutilized regional resource for future nutraceutical and anticancer research. Furthermore, individual bioactive compounds will be isolated from Bidah pomegranate materials and evaluated against a broad spectrum of cancer cell lines.

## Figures and Tables

**Table 1:** HPLC data for the peaks obtained from B6 fraction at 280 nm.

Peak	RT (min)	Width (min)	Area (mAU*s)	Height (mAU)	Area (%)
1	3.377	0.0992	53.28	7.85	9.74
2	7.225	0.2080	68.13	4.37	12.45
3	7.577	0.1892	249.85	17.46	45.66
4	8.158	0.0844	19.66	3.56	3.59
5	8.255	0.0969	26.97	4.09	4.93
6	8.602	0.1452	109.47	10.90	20.00
7	9.369	0.0994	19.89	3.16	3.63

**Table 2:** GC–MS-identified phytochemicals with relevance to mitochondrial dysfunction and apoptosis.

Compound Name	RT (min)	Area (%)	Link to Intrinsic Apoptosis	Reference
Falcarinol	12.9	8.1	Mitochondrial depolarization and intrinsic apoptosis	[14]
Estra-1,3,5(10)-trien-17β-ol	27.3	8.38	Steroid-mediated modulation of mitochondrial signaling	[15]
Hexadecanoic acid, methyl ester	26.65	18.43	Associated with lipid metabolic and mitochondrial stress	[16]
Oleic acid	28.98	4.18	Alters mitochondrial membrane composition and ΔΨm	[17]
Octadecanoic acid, 4-hydroxy-, methyl ester	33.73	6.6	Potential oxidative and mitochondrial stress modulation	[18]
Cedran-diol (8S,14-)	37.57	1.83	Associated with mitochondrial dysfunction and apoptosis sensitization	[19]

**Table 3:** Primer sequence.

Gene	Forward Primer (5′-3′)	Reverse Primer (5′-3′)	Reference
*SKP2*	GCTGCTAAAGGTCTCTGGTGT	AGGCTTAGATTC TGCAACTTG	[31]
*CDKN1B*	TGGAGAAGCACTGCAGAGAC	GCGTGTCCTCAGAGTTAGCC	[32]
*PTGS2*	TGAAGAACTTACAGGAGAAAA	TACCAGAAGGGCAGGATACA	[33]
*CAT*	CCAGAAGAAAGCGGTCAAGAA	GAGATCCGGACTGCACAAAG	[34]
*PARP1*	TTCAACAAGCAGCAAGTGCC	CCTTTGGGGTTACCCACTCC	[35]
*BCL2L1*	TTACCTGAATGACCACCTA	ATTTCCGACTGAAGAGTGA	[36]
*BID*	GCTGTATAGCTGCTTCCAGTGTA	GCTATCTTCCAGCCTGTCTTCTC	[37]
GAPDH	TCAACGACCACTTTGTCAAGCTCA	GCTGGTGGTCCAGGGGTCTTACT	[NM_002046.7]
*CASP3*	ATGGTTTGAGCCTGAGCAGA	GGCAGCATCATCCACACATAC	[38]

## Data Availability

The original contributions presented in this study are included in the article. Further inquiries can be directed to the corresponding author.

## Abbreviations

The following abbreviations are used in this manuscript:

B6	Ethyl acetate/methanol fraction of Bidah pomegranate
SGCC	Silica gel column chromatography
AO/PI	Acridine orange/propidium iodide
DPPH	2,2-diphenyl-1-picrylhydrazyl
TLC	Thin-layer chromatography
